# Prolonged Mechanical Ventilation and In-Hospital Mortality Following Open-Heart Surgery: A Retrospective Cohort Study

**DOI:** 10.14740/cr2269

**Published:** 2026-08-31

**Authors:** Jofinda Virsty, Muhammad Budi Kurniawan, Radian Ahmad Halimi, Dian Nuryanda, Reza Widianto Sudjud, Indriasari, Jenifer Kiem Aviani

**Affiliations:** a Faculty of Medicine, Universitas Padjadjaran, Bandung, West Java, Indonesia; b Cardiothoracic and Vascular Division, Intensive Care Division, Department of Anesthesiology and Intensive Care, Dr. Hasan Sadikin General Hospital, Padjadjaran University, Bandung, West Java, Indonesia; c Subdivision of Neuroanesthesia and Critical Care, Department of Anesthesiology and Intensive Therapy, Dr. Hasan Sadikin General Hospital, Padjadjaran University, Bandung, West Java, Indonesia; d Department of Anesthesiology and Intensive Therapy, Dr. Hasan Sadikin General Hospital, Padjadjaran University, Bandung, West Java, Indonesia

**Keywords:** Open-heart surgery, Prolonged mechanical ventilation, In-hospital mortality, Risk factors, Receiver operating characteristics, Binary logistic regression

## Abstract

**Background:**

Prolonged mechanical ventilation (PMV) after open-heart surgery is associated with adverse postoperative outcomes, but evidence regarding its association with mortality in Indonesia remains limited. This study evaluated the association between PMV and in-hospital mortality and identified factors associated with PMV.

**Methods:**

A retrospective cohort study was conducted among adults undergoing open-heart surgery at Dr. Hasan Sadikin General Hospital, Bandung, Indonesia, between 2023 and 2024. PMV was defined using two thresholds (> 24 h and ≥ 96 h). Factors associated with mortality were evaluated using univariable and multivariable logistic regression.

**Results:**

A total of 249 patients were included, with an in-hospital mortality rate of 18.9%. PMV occurred in 62.2% (> 24 h) and 5.6% (≥ 96 h) of patients. In multivariable analysis, PMV > 24 h (adjusted odds ratio (aOR) 3.259, 95% confidence interval (CI) 1.438–7.386; P = 0.005) and longer operative duration (aOR 1.009, 95% CI 1.004–1.015; P = 0.001) were associated with in-hospital mortality after adjustment. Using the ≥ 96-h definition, PMV (aOR 15.422, 95% CI 4.112–57.844; P < 0.001), operative duration (aOR 1.009, 95% CI 1.003–1.014; P = 0.002), and age (aOR 1.037, 95% CI 1.000–1.075; P = 0.049) remained associated with in-hospital mortality after adjustment. No statistically significant factors associated with PMV were identified.

**Conclusions:**

PMV was associated with increased in-hospital mortality following open-heart surgery, with a stronger association for ventilation ≥ 96 h. Longer operative duration also predicted mortality, highlighting the importance of perioperative risk stratification and optimized postoperative ventilatory management.

## Introduction

Cardiovascular disease remains the leading cause of mortality worldwide, accounting for approximately 17.9 million deaths annually [1]. As its prevalence continues to increase, the demand for open-heart surgery has also risen, with more than two million procedures performed each year [2]. Despite advances in perioperative care, cardiac surgery remains associated with significant postoperative morbidity and mortality [3, 4].

Mechanical ventilation is essential for postoperative recovery after cardiac surgery. While most patients can be extubated within 1–6 h, approximately 20% require prolonged mechanical ventilation (PMV) [5, 6]. PMV has been associated with prolonged intensive care unit (ICU) and hospital stays, increased healthcare costs, postoperative complications, and higher mortality [4, 5, 7]. Several patient- and procedure-related factors, including age, sex, comorbidities, and type of surgery, have been reported to influence the risk of PMV [3, 8–11].

Most evidence originates from countries with healthcare systems that differ from Indonesia. Local data remain limited and inconsistent. A study at Dr. Hasan Sadikin General Hospital, Bandung (2014–2016), reported that 69.7% of 175 patients required PMV [12], whereas a study at Dr. Cipto Mangunkusumo National General Hospital, Jakarta (2016–2020), found an incidence of 28.25% among 315 patients [13]. However, these studies primarily focused on the incidence and predictors of PMV, with limited evaluation of its association with postoperative mortality. Therefore, this study aimed to evaluate the association between prolonged mechanical ventilation and in-hospital mortality following open-heart surgery, while also identifying demographic, clinical, and operative factors associated with PMV at Dr. Hasan Sadikin General Hospital, Bandung, between 2023 and 2024.

## Materials and Methods

### Ethical declarations

This study was approved by the Research Ethics Committee of Padjadjaran University (No. 950/UN6.KEP/EC/2025) and the Research Ethics Committee of Dr. Hasan Sadikin General Hospital (No. DP.04.03/D.XIV.4.4/3166/2025). The study was conducted in accordance with the Declaration of Helsinki and applicable institutional regulations. As this retrospective study utilized anonymized medical record data, patient confidentiality was strictly maintained throughout the study.

### Study design

This retrospective cohort study was conducted using secondary data obtained from the medical records of Dr. Hasan Sadikin General Hospital, Bandung, Indonesia. The study included adult patients who underwent open-heart surgery between January 2023 and December 2024. The primary outcome was in-hospital mortality, while PMV, defined using two predefined thresholds (> 24 h and ≥ 96 h), was evaluated as the primary exposure.

### Inclusion and exclusion criteria

Eligible participants were adults aged ≥ 18 years who underwent open-heart surgery during the study period and had complete medical records containing demographic, clinical, operative, ventilatory, and outcome data. Patients with incomplete records for any study variable were excluded from the analysis.

Patients were also excluded if they were transferred to another hospital before completion of postoperative care or if they died intraoperatively.

### Data extraction

Total sampling was employed, whereby all eligible patients meeting the inclusion and exclusion criteria were included. Data were extracted from medical records using a standardized data collection form. Variables collected included age, sex, comorbidities (diabetes mellitus, hypertension, chronic kidney disease, obesity, dyslipidemia, and smoking status), type of surgery (coronary artery bypass grafting (CABG) or valve replacement), duration of surgery, prolonged mechanical ventilation (> 24 h and ≥ 96 h), and in-hospital mortality.

Mechanical ventilation duration was categorized using two predefined thresholds. A duration of > 24 h was selected as the primary clinical definition of PMV based on the Society of Thoracic Surgeons (STS) quality metrics and previous cardiac surgery studies, in which mechanical ventilation beyond 24 h is considered a major postoperative morbidity [14]. A duration of ≥ 96 h was additionally evaluated because it corresponds to the ICD-10 Procedure Coding System (ICD-10-PCS) classification for respiratory ventilation lasting more than 96 consecutive hours code 5A1955Z, a definition widely used in administrative databases and hospital coding systems.

### Statistical analysis

Statistical analyses were performed using IBM SPSS Statistics for Windows, version 20.0 (IBM Corp., Armonk, NY, USA).

### Bivariate analysis

Continuous variables were assessed for normality using the Shapiro–Wilk test and are presented as mean ± standard deviation (SD) or median with interquartile range (IQR), as appropriate. Categorical variables are presented as frequencies and percentages.

Baseline characteristics were compared between survivors and non-survivors using the independent *t*-test or Mann–Whitney U test for continuous variables and the Chi-square test or Fisher’s exact test for categorical variables, as appropriate. Receiver operating characteristic (ROC) curve analysis was performed to evaluate the ability of continuous variables (age and duration of surgery) to predict in-hospital mortality and PMV. Separate ROC curves were constructed for mortality, PMV ≥ 24 h, and PMV ≥ 96 h. The area under the curve (AUC) with 95% confidence intervals (CIs) was calculated, and the optimal cut-off values were determined using the Youden index. Sensitivity, specificity, positive predictive value, and negative predictive value at the optimal cut-off were also reported.

### Univariable and multivariable logistic regression

Variables with a bivariate P-value < 0.20 were subsequently entered into univariable logistic regression to estimate crude odds ratios (ORs) and 95% CIs. Variables demonstrating a univariable P-value < 0.20 were then included in multivariable logistic regression models to evaluate factors associated with in-hospital mortality and PMV after adjustment for other variables in the model. Two separate multivariable models were constructed to evaluate the association between PMV, defined as ≥ 24 h and ≥ 96 h, and in-hospital mortality to avoid multicollinearity between the two PMV definitions. Additional multivariable logistic regression analyses were performed to evaluate factors associated with PMV (> 24 h and ≥ 96 h). Crude and adjusted ORs with 95% CIs were reported.

To reduce the risk of model overfitting, the number of predictors included in each multivariable model was limited according to the events-per-variable (EPV) principle, with at least 10 outcome events per predictor variable. Multicollinearity among independent variables was assessed using the variance inflation factor (VIF), with a VIF > 5 indicating significant multicollinearity. Model calibration was evaluated using the Hosmer–Lemeshow goodness-of-fit test. All statistical tests were two-sided, and a P-value < 0.05 was considered statistically significant.

## Results

### ROC curve analysis of continuous variables

ROC curve analysis showed that both age and duration of surgery were significantly associated with in-hospital mortality but demonstrated limited discriminatory ability. Age yielded an AUC of 0.622 (95% CI: 0.533–0.712, P = 0.009), with an optimal cut-off of 61.5 years (sensitivity 51.1%, specificity 69.8%). Duration of surgery had an AUC of 0.633 (95% CI: 0.541–0.726, P = 0.004), with an optimal cut-off of 280.5 min (sensitivity 38.3%, specificity 86.1%). In contrast, neither variable significantly predicted PMV defined as ≥ 24 h or ≥ 96 h (all AUCs ≤ 0.588, all P >0.05), and therefore no optimal cut-off values were identified for these outcomes (Table 1).

**Table 1:** ROC Curve Analysis of Continuous Variable Toward In-Hospital Mortality and PMV

Outcome	Predictor	AUC (95% CI)	P-value	Optimal cut-off	Sensitivity (%)	Specificity (%)
Mortality	Age	0.622 (0.533 to 0.712)	0.009*	61.5 (∼62)	51.1	69.8
	Duration of surgery	0.633 (0.541 to 0.726)	0.004*	280.50 (∼280)	38.3	86.1
PMV > 24-h	Age	0.528 (0.455 to 0.601)	0.461	-	-	-
	Duration of surgery	0.512 (0.437 to 0.587)	0.751	-	-	-
PMV ≥ 96-h	Age	0.554 (0.387 to 0.721)	0.498	-	-	-
	Duration of surgery	0.588 (0.424 to 0.752)	0.268	-	-	-

*Statistically significant results (P < 0.05). AUC: area under the curve; CI: confidence interval; PMV: prolonged mechanical ventilation.

### Baseline characteristics and bivariate analysis toward in-hospital mortality

Baseline characteristics according to in-hospital mortality are presented in Table 2. A total of 249 patients were included, with an overall in-hospital mortality rate of 18.9% (47/249). The median age was 57.0 years (IQR 13.0), and most patients were male (71.1%). Hypertension was the most common comorbidity (44.6%), followed by diabetes mellitus (27.7%) and smoking (27.3%). Coronary artery bypass grafting (CABG) was the predominant surgical procedure (69.5%), while the median duration of surgery was 225.0 min (IQR 79.5). Overall, 62.2% and 5.6% of patients required PMV for ≥ 24 h and ≥ 96 h, respectively.

**Table 2:** Baseline Characteristic and Bivariate Analysis Toward In-Hospital Mortality

Variable	Total (n = 249)	Survivor (n = 202)	Non-survivor (n = 47)	P-value
Age (years) (median (IQR))	57.00 (13.00)	56.00 (12.00)	62.00 (10.00)	0.009*
< 62 years^a^	164 (65.9%)	141 (69.8%)	23 (48.9%)	0.006*
≥ 62 years	85 (34.1%)	61 (30.2%)	24 (51.1%)	
Sex				
Male (%)	177 (71.1%)	143 (70.8%)	34 (72.3%)	1.000
Female (%)	72 (28.9%)	59 (29.2%)	13 (27.7%)	
Comorbidities				
Diabetes (%)	69 (27.7%)	58 (28.7%)	11 (23.4%)	0.295
Hypertension (%)	111 (44.6%)	87 (43.1%)	24 (51.1%)	0.203
Obesity (%)	29 (11.6%)	24 (11.9%)	5 (10.6%)	0.552
Dyslipidemia (%)	33 (13.3%)	33 (16.3%)	0 (0.0%)	0.001*
Renal failure (%)	25 (10.0%)	17 (8.4%)	8 (17.0%)	0.072
Smoking (%)	68 (27.3%)	54 (26.7%)	14 (29.8%)	0.398
Type of surgery				
Valve replacement (%)	72 (28.9%)	58 (28.7%)	14 (29.8%)	0.734
Single (AV/MV) (%)	56 (22.5%)	47 (23.3%)	9 (19.1%)	
Double (AV + MV/MV + TV) (%)	13 (5.2%)	9 (4.5%)	4 (8.5%)	
Triple (AV + MV + TV) (%)	3 (1.2%)	2 (1%)	1 (2.1%)	
CABG (%)	173 (69.5%)	141 (69.8%)	32 (68.1%)	
Mix (CABG + AV/CABG + MV + TV) (%)	4 (1.6%)	3 (1.5%)	1 (2.1%)	
Duration of surgery (min) (median (IQR))	225.00 (79.50)	225.00 (84.00)	244.00 (110.00)	0.004*
< 280 min	203 (81.5%)	174 (86.1%)	29 (61.7%)	< 0.0001*
≥ 280 min	46 (18.5%)	28 (13.9%)	18 (38.3%)	
Prolonged mechanical ventilation				
24-h cut-off				
≤ 24-h (%)	94 (37.8%)	85 (42.1%)	9 (19.1%)	0.002*
> 24-h (%)	155 (62.2%)	117 (57.9%)	38 (80.9%)	
96-h cut-off				
< 96-h (%)	235 (94.4%)	198 (98%)	37 (78.7%)	0.000*
≥ 96-h (%)	14 (5.6%)	4 (2%)	10 (21.3%)	

^a^Cut-off was determined from ROC curve analysis. *Statistically significant results (P < 0.05). AV: aortic valve; CABG: coronary artery bypass grafting; IQR: interquartile range; MV: mitral valve; TV: tricuspid valve.

Non-survivors were significantly older than survivors (median 62.0 (IQR 10.0) vs. 56.0 (IQR 12.0) years, P = 0.009). Consistent with the ROC analysis (Table 1), patients aged ≥ 62 years had a significantly higher mortality than those aged < 62 years (51.1% vs. 30.2%, P = 0.006). Similarly, non-survivors had a longer duration of surgery than survivors (median 244.0 (IQR 110.0) vs. 225.0 (IQR 84.0) min, P = 0.004), and a significantly greater proportion underwent procedures lasting ≥ 280 min, the optimal cut-off identified by ROC analysis (38.3% vs. 13.9%, P < 0.001).

Among comorbidities, dyslipidemia was less frequent in non-survivors than survivors (0.0% vs. 16.3%, P = 0.001), whereas renal failure showed a non-significant trend toward a higher prevalence in non-survivors (17.0% vs. 8.4%, P = 0.072). No significant differences were observed for sex, diabetes mellitus, hypertension, obesity, smoking status, or type of surgery (all P > 0.05). PMV was significantly associated with mortality, with non-survivors more frequently requiring ventilation for ≥ 24 h (80.9% vs. 57.9%, P = 0.002) and ≥ 96 h (21.3% vs. 2.0%, P < 0.001).

### Bivariate analysis toward PMV

Baseline characteristics according to PMV are summarized in Table 3. With the 24-h cut-off, diabetes mellitus was the only variable significantly associated with PMV, occurring more frequently in patients requiring ventilation for ≥ 24 h than in those ventilated for < 24 h (32.3% vs. 20.2%, P = 0.027). No significant associations were identified for age, sex, other comorbidities, type of surgery, or duration of surgery. Likewise, no demographic, clinical, or operative variables were significantly associated with PMV when the 96-h cut-off was applied (all P > 0.05).

**Table 3:** Bivariate Analysis Toward Prolonged Mechanical Ventilation

Variable	24-h cut-off			96-h cut-off		
	≤ 24-h (n = 94)	> 24-h (n = 155)	P-value	< 96-h (n = 235)	≥ 96-h (n = 14)	P-value
Age (median (IQR))	57.00 (12.25)	58.00 (14.00)	0.460	57.00 (13.00)	60.50 (13.50)	0.498
Sex						
Male (%)	71 (75.5%)	106 (68.4%)	0.144	167 (71.06%)	10 (71.43%)	0.621
Female (%)	23 (24.5%)	49 (31.6%)		68 (28.94%)	4 (28.57%)	
Comorbidities						
Diabetes (%)	19 (20.2%)	50 (32.3%)	0.027*	66 (28.09%)	3 (21.43%)	0.424
Hypertension (%)	44 (46.8%)	67 (43.2%)	0.337	106 (45.11%)	5 (35.71%)	0.344
Obesity (%)	12 (12.8%)	17 (11.0%)	0.406	28 (11.91%)	1 (7.14%)	0.498
Dyslipidemia (%)	16 (17.0%)	17 (11.0%)	0.121	32 (13.62%)	1 (7.14%)	0.422
Renal failure (%)	11 (11.7%)	14 (9.0%)	0.318	24 (10.21%)	1 (7.14%)	0.579
Smoking (%)	25 (26.6%)	43 (27.7%)	0.482	62 (26.38%)	6 (42.86%)	0.150
Type of surgery						
Valve replacement (%)	26 (27.7%)	46 (29.7%)		66 (28.09%)	6 (42.86%)	0.736
Single (AV/MV) (%)	21 (22.3%)	35 (22.6%)		51 (21.7%)	5 (35.71%)	
Double (AV + MV/MV + TV) (%)	4 (4.3%)	9 (5.8%)		12 (5.11%)	1 (7.14%)	
Triple (AV + MV + TV) (%)	1 (1.1%)	2 (1.3%)	0.958	3 (1.28%)	0 (0%)	
CABG (%)	67 (71.3%)	106 (68.4%)		165 (70.21%)	8 (57.14%)	
Mix (CABG + AV/CABG + MV + TV) (%)	1 (1.1%)	3 (1.9%)		4 (1.7%)	0 (0%)	
Duration of surgery (min) (median (IQR))	223.50 (85.50)	226.00 (75.00)	0.751	225.00 (74.00)	230.00 (123.25)	0.267

*Statistically significant results (P < 0.05). AV: aortic valve; CABG: coronary artery bypass grafting; IQR: interquartile range; MV: mitral valve; TV: tricuspid valve.

### Univariable and multivariable analysis of factors associated with in-hospital mortality

As bivariate analysis identified only diabetes mellitus as significantly associated with PMV using the 24-h definition and no significant factors using the 96-h definition (Table 3), multivariable logistic regression was not performed for PMV. Instead, multivariable logistic regression analyses were conducted to evaluate the association between PMV and in-hospital mortality after adjustment for variables included in the models (Table 4).

**Table 4:** Univariable and Multivariable Logistic Regression Analysis of Factors Associated With In-Hospital Mortality

Variable	Crude OR (95% CI)	P-value	Adjusted OR (95% CI)	P-value
Model 1				
Age (years)	1.036 (1.002 to 1.072)	0.039*	1.030 (0.994 to 1.068)	0.105
Duration of surgery (min)	1.009 (1.004 to 1.015)	< 0.0001*	1.009 (1.004 to 1.015)	0.001*
Renal failure	2.232 (0.900 to 5.538)	0.083	2.201 (0.825 to 5.871)	0.115
PMV > 24-h	3.067 (1.408 to 6.681)	0.005*	3.259 (1.438 to 7.386)	0.005*
Model 2				
Age (years)	1.036 (1.002 to 1.072)	0.039*	1.037 (1.000 to 1.075)	0.049*
Duration of surgery (min)	1.009 (1.004 to 1.015)	< 0.0001*	1.009 (1.003 to 1.014)	0.002*
Renal failure	2.232 (0.900 to 5.538)	0.083	2.188 (0.820 to 5.838)	0.118
PMV ≥ 96-h	13.378 (3.983 to 44.932)	< 0.0001*	15.422 (4.112 to 57.844)	< 0.0001*

*Statistically significant results (P < 0.05). Model 1: Model diagnostics: Omnibus test P < 0.0001; Hosmer–Lemeshow goodness-of-fit P = 0.859; Nagelkerke R^2^ = 0.170. No evidence of multicollinearity was observed (all VIF < 2.0). Model 2: Model diagnostics: Omnibus test P < 0.0001; Hosmer–Lemeshow goodness-of-fit P = 0.225; Nagelkerke R^2^ = 0.227. No evidence of multicollinearity was observed (all VIF < 2.0).

In the model using the 24-h PMV definition (model 1), longer duration of surgery (adjusted OR (aOR) 1.009, 95% CI: 1.004–1.015, P = 0.001) and PMV ≥ 24 h (aOR 3.259, 95% CI: 1.438–7.386, P = 0.005) were associated with higher odds of in-hospital mortality after adjustment, whereas age and renal failure were not significant after adjustment. In the model using the 96-h PMV definition (model 2), age (aOR 1.037, 95% CI: 1.000–1.075, P = 0.049), duration of surgery (aOR 1.009, 95% CI: 1.003–1.014, P = 0.002), and PMV ≥ 96 h (aOR 15.422, 95% CI: 4.112–57.844, P < 0.001) remained associated with in-hospital mortality, while renal failure was not significantly associated with mortality. Both models demonstrated good calibration according to the Hosmer–Lemeshow goodness-of-fit test (model 1: P = 0.859; model 2: P = 0.225), with no evidence of multicollinearity (all VIF < 2.0).

## Discussion

The present study demonstrated an in-hospital mortality rate of 18.9% among patients undergoing open-heart surgery. This rate is substantially higher than the pooled in-hospital mortality of 2.38% reported in a systematic review and meta-analysis of more than 614,000 patients undergoing major cardiac surgery across the Asia–Oceania region, where isolated CABG procedures had a mortality of 1.97% and valve surgery 3.97% [15].

Although direct comparisons should be interpreted cautiously because of differences in patient characteristics, case complexity, and hospital referral patterns, the higher mortality observed in our cohort may partly reflect the challenges of delivering cardiac surgical care in lower-middle-income countries. Limited access to specialized cardiac surgical services often results in delayed presentation, more advanced disease, and a greater burden of perioperative risk at the time of surgery. Moreover, disparities in infrastructure, workforce capacity, intensive care resources, and financial accessibility may further influence postoperative outcomes. Nevertheless, successful cardiac surgery programs in several low- and middle-income countries have demonstrated that outcomes comparable to those of high-income countries are achievable when sustainable surgical infrastructure, multidisciplinary perioperative care, and appropriate resource allocation are established [16, 17].

The study population was predominantly male (71.1%), with a median age of 57 years. Hypertension was the most prevalent comorbidity, affecting nearly half of the patients, followed by diabetes mellitus and smoking. CABG was the most frequently performed procedure, accounting for approximately 70% of all surgeries. These findings are consistent with the epidemiology of cardiovascular disease, where coronary artery disease remains more prevalent in men and hypertension is the leading modifiable cardiovascular risk factor. CABG continues to represent the most common cardiac surgical procedure worldwide because coronary artery disease accounts for the majority of patients requiring surgical revascularization. The predominance of CABG in our cohort therefore reflects the current burden of ischemic heart disease rather than differences in surgical practice [6].

The principal finding of this study was that PMV was associated with higher odds of in-hospital mortality after adjustment for the variables included in the models, regardless of the definition used. Patients requiring mechanical ventilation for ≥ 24 h had more than threefold higher adjusted odds of mortality, whereas those requiring ventilation for ≥ 96 h had more than 15-fold higher adjusted odds of mortality. These findings are consistent with the recent systematic review and meta-analysis by Wang et al, which included over 68,000 cardiac surgery patients and demonstrated that PMV was associated with a markedly increased risk of in-hospital mortality (pooled OR 14.13, 95% CI 12.16–16.41) [6]. Similarly, contemporary observational studies have consistently shown that patients requiring PMV experience substantially higher mortality, prolonged ICU and hospital stays, and greater healthcare utilization [18–20].

Patients requiring prolonged ventilatory support are likely to have greater underlying disease severity and more severe postoperative complications, including respiratory failure, myocardial dysfunction, hemodynamic instability, infection, acute kidney injury, and neurological impairment, all of which may contribute substantially to mortality [18–22]. Thus, PMV may primarily serve as a marker of persistent organ dysfunction and greater postoperative illness severity rather than a direct cause of mortality. Because these factors were not comprehensively captured in our retrospective dataset, the observed association between PMV and mortality may partly reflect the severity of the underlying disease and postoperative clinical condition. The substantially stronger association observed with the ≥ 96-h definition may therefore reflect a subgroup with more severe and prolonged postoperative illness and should not be interpreted as evidence of a causal effect of ventilation duration. Accordingly, the association observed with both PMV definitions should be interpreted in the context of underlying disease severity and postoperative complications.

Besides PMV, operative duration remained a factor associated with in-hospital mortality in both multivariable models. Longer operative times likely reflect greater surgical complexity and prolonged exposure to cardiopulmonary bypass, aortic cross-clamping, anesthesia, and systemic inflammatory activation, all of which contribute to myocardial injury, pulmonary dysfunction, coagulopathy, and multiorgan impairment [23]. Previous studies have likewise identified prolonged operative or cardiopulmonary bypass duration as important predictors of adverse outcomes following cardiac surgery [24].

Increasing age is a well-established predictor of adverse outcomes following cardiac surgery. In the present study, age remained independently associated with in-hospital mortality only in the model using the ≥ 96-h PMV definition, suggesting that its effect may be partly mediated through postoperative complications, including prolonged ventilatory support and operative complexity. ROC analysis identified an optimal age cut-off of 62 years, which is considerably lower than the 75-year threshold proposed by Afilalo et al based on 30-day operative mortality in a large multicenter cardiac surgery cohort. This discrepancy may reflect differences in patient characteristics, healthcare settings, and outcome definitions, as patients in low- and middle-income countries often present with more advanced disease and greater untreated comorbidity at a younger age. Afilalo et al further suggested that the adverse effect of aging is primarily driven by the accumulation of comorbidities and frailty, which substantially reduce physiological reserve and increase vulnerability to surgical stress. Although frailty was not evaluated in the present study, it may partly explain the higher mortality observed among older patients undergoing open-heart surgery [25].

Renal failure showed a trend toward increased in-hospital mortality but did not remain significant after multivariable adjustment, possibly because of the limited number of affected patients and overlapping effects with other perioperative factors. Nevertheless, renal dysfunction is a well-established predictor of adverse outcomes after cardiac surgery, as impaired renal function reduces physiological reserve and promotes inflammation, endothelial dysfunction, and multiorgan injury. Previous study has also demonstrated that pre-existing chronic kidney disease amplifies the adverse impact of postoperative renal dysfunction on mortality [26]. Therefore, the lack of statistical significance in our study should be interpreted cautiously and may reflect limited statistical power rather than the absence of a true association.

Contrary to our initial hypothesis, no demographic, clinical, or operative variables independently predicted PMV. Although diabetes mellitus was associated with PMV using the 24-h definition in the bivariate analysis, this association was not sufficiently robust to warrant multivariable modeling and was not observed using the 96-h definition. Likewise, ROC analysis demonstrated that neither age nor operative duration showed meaningful discriminatory ability for predicting PMV. These findings differ from those of previous studies, which have identified advanced age, female sex, chronic renal failure, reduced left ventricular ejection fraction, obesity, chronic obstructive pulmonary disease, prolonged cardiopulmonary bypass time, and previous cardiac surgery as important predictors of PMV. The discrepancy may be explained by the limited number of variables available in the present retrospective study. Several well-established determinants of PMV—including cardiopulmonary bypass duration, aortic cross-clamp time, left ventricular function, transfusion requirements, postoperative bleeding, and respiratory complications—were unavailable in our dataset and therefore could not be evaluated [27].

Overall, the present findings reinforce PMV as an important marker of adverse postoperative outcomes following open-heart surgery. Patients requiring prolonged ventilatory support may be considered a high-risk population warranting closer monitoring and individualized perioperative management. These findings support the implementation of multidisciplinary postoperative care involving cardiac surgeons, anesthesiologists, intensivists, respiratory therapists, and nurses, together with standardized ventilation and extubation protocols to facilitate timely weaning when clinically appropriate. Such strategies are consistent with enhanced recovery after surgery (ERAS) recommendations, which emphasize protocolized perioperative care, early mobilization, and early extubation to improve postoperative recovery. In addition, incorporating preoperative risk stratification into perioperative pathways may facilitate the early identification of patients at risk for PMV and adverse outcomes [28–31]. Future multicenter prospective studies incorporating comprehensive perioperative variables are warranted to develop and validate robust prediction models and to determine whether protocolized early extubation strategies can safely reduce PMV and improve postoperative outcomes.

### Conclusion

PMV is a factor associated with in-hospital mortality following open-heart surgery, with a stronger association observed for ventilation lasting ≥ 96 h. This association may partly reflect underlying disease severity and postoperative complications. Together with longer operative duration, these findings emphasize the importance of perioperative risk stratification and optimized postoperative ventilatory management to improve clinical outcomes.

## Data Availability

The datasets generated and/or analyzed during the current study are not publicly available because they contain confidential patient information but are available from the corresponding author upon reasonable request and with permission from Dr. Hasan Sadikin General Hospital.

## Abbreviations

AVR: aortic valve replacement
CABG: coronary artery bypass grafting
ERAS: enhanced recovery after surgery
ICD: International Statistical Classification of Diseases and Related Health Problems
ICU: intensive care unit
LOS: length of stay
MVR: mitral valve replacement
PMV: prolonged mechanical ventilation
TVR: tricuspid valve replacement
